# Assessment of health-related quality of life among Afghan refugees in Quetta, Pakistan

**DOI:** 10.1371/journal.pone.0288834

**Published:** 2024-02-01

**Authors:** Shoaib Kaleem, Tawseef Ahmad, Abdul Wahid, Hamad Haider Khan, Tauqeer Hussain Mallhi, Yaser Mohammed Al-Worafi, Anila Alam, Asad Khan, Yusra Habib Khan, Faiz Ullah Khan

**Affiliations:** 1 Department of Pharmacy Practice, Faculty of Pharmacy and Health Sciences, University of Balochistan, Quetta, Pakistan; 2 Department of Clinical Pharmacy, Faculty of pharmaceutical sciences, Prince of Songkla University, Hat-Yai, Thailand; 3 Department of Endocrinology, First Affiliated Hospital of Xian Jiaotong University, Xi’an, China; 4 Department of Clinical Pharmacy, College of Pharmacy, Jouf University, Sakaka, Saudi Arabia; 5 College of Pharmacy, University of Science and Technology of Fujairah, Fujariah, United Arab Emirates; 6 Department of Pharmacy, Sardar Bahadur Khan Women University Balochistan, Quetta, Pakistan; 7 Discipline of Clinical Pharmacy, School of Pharmaceutical Sciences, Universiti Sains Malaysia, George Town, Penang, Malaysia; 8 Department of Pharmacy Administration and Clinical Pharmacy, School of Pharmacy, Xi’an Jiaotong University, Xi’an, China; Access Alliance Multicultural Health and Community Services: Access Alliance, CANADA

## Abstract

The study aims to assess the health-related Quality of Life (HRQOL) and its association with socio-demographic factors among the Afghan refugees residing in Quetta, Pakistan. For this purpose, a cross-sectional, descriptive study design by adopting Euro QOL five dimensions questionnaire (EQ-5D) for the assessment of HRQOL was conducted by approaching Afghan refugees from the camp and other areas of Quetta, Pakistan. Furthermore, this study also involved descriptive analysis to expound participant’s demographic characteristics while inferential statistics (Kruskal-Wallis and Mann–Whitney test, P < 0.05) were used to compare EQ-5D scale scores. All analyses were performed using SPSS v 20. Herein, a total of 729 participants were enrolled and were subsequently (n = 246, 33.7%) categorized based on their age of 22–31 years (31.30 ± 15.40). The results of mean EQ-5D descriptive score (0.85 ± 0.20) and EQ-VAS score (78.60 ± 11.10) indicated better HRQOL in the current study respondents as compared to studies conducted in other refugee camps around the globe. In addition, demographic characteristics including age, marital status, locality, years of living as refugees, life as a refugee residing out of Pakistan, place of residence in Afghanistan, educational qualification, occupation, and arrested for crime were the statistically significant predictors (*P* < 0.05) of EQ-5D index scores. However, gender, living status, monthly income, preferred place of treatment were non-significant predictors (*P* > 0.05). The results of current study provided evidence for a model that correlated with participant’s socio-demographic information and HRQOL. Moreover, this study also revealed a baseline assessment for the health status of Afghan refugees, interestingly, these results could be applied for improving HRQOL of the given participants. In conclusion, the HRQOL of Afghan refugees residing in Quetta, Pakistan can largely be improved by providing adequate healthcare facilities, education and employment opportunities, mental and social support, and providing adequate housing and basic necessities of life.

## Introduction

A refugee is a person fleeing life-threatening conditions. In daily parlance and for journalistic purposes, this is roughly the meaning of "refugee hood." Predictably, in legal and political circles, among those officials who formulate refugee policies for states and international agencies, the meaning is considerably more circumscribed [1]. Worldwide, the number of refugees and asylum seekers is estimated to be about 11.5 million. Plus a much larger number of former refugees who have obtained a residence permit in a new country [2]. Among migrants fleeing war-torn areas, potentially traumatic events like close-quarters combat are frequent [3, 4], and many refugees may have mental illness as a result of these events [3, 5, 6] Everyone has the right to seek and receive asylum from persecution in other countries [7]. International assistance to refugees is channeled through United Nations High Commissioner for Refugees (UNHCR), through Non-Governmental Organizations (NGOs) and bilaterally. UNHCR is one of the few UN agencies that depends almost entirely on voluntary contributions to finance its operations. Less than two percent of UNHCR’s annual budget comes from the United Nations; the rest is contributed by states, individuals, and the private sector [8]. Pakistan is neither a party to the 1951 Convention relating to the Status of Refugees nor the 1967 Protocol relating to the Status of Refugees [9]. The cost of refugee relief to Pakistan in the mid-1980s was about 1 million dollars a day, all of which was financed by contributions from foreign governments (channeled through UNHCR) and private voluntary organizations [10]. More than $273 million in humanitarian aid has already been given by the US to Pakistan’s host communities and Afghan refugees there. The US donated approximately $60 million to the Pakistani host communities and Afghan refugees for the fiscal year 2022 [11]. Afghans in Pakistan are in some measure both refugees and economic migrants; they may face political threats in Afghanistan but are also working to support relatives there [12]. Migration and subsequent adjustment cause stress, which increases the risk of sickness [13]. Afghans comprise the largest refugee population in the world [14]. In general, social networks and support are seen as significant protective factors for both physical and mental health [15]. Pakistan has received Afghan refugees since the 1980s. There are 18 camps for Afghan refugees located in five districts of Balochistan Province. Six of the 18 camps are labeled as “new camps” and were established in response to the refugee influx in November 2001 [16]. The remaining 12 “old camps” cater to Afghan refugees who have been arriving since the time of the Russian invasion of Afghanistan in 1979. The number of Afghans in Pakistan is 3,049,268 individuals, as enumerated in the Census of Afghans in Pakistan 2005. Almost half reside in five districts (20.1 percent in Peshawar, 11.1 percent in Quetta, 7.6% in Nowshera, 5.1% in Pishin, and 4.3% in Karachi), while the balance is spread across the remaining 120 districts of Pakistan. According to the Census, there are 769,268 (25.2 percent) Afghans, or 115,565 families, in Balochistan province. The 2005 Census notes that if Pakistan’s population ratios have not changed since the 1998 Census, then Afghans in Balochistan would be equivalent to 10 percent of Pakistanis, and in the Northwest Frontier Province (NWFP) the figure would be 7.6 percent [17]. Following the American invasion of Afghanistan, the number of refugees has increased in recent years. [18]. While in Pushtun-dominated areas, Afghan Pashtuns were allowed to settle [10]. Quetta district ranks second in Pakistan among the top ten districts with Afghans under the age of five (11.6 percent) [17]. In the last few decades, there has been an enormous amount of research on quality of life (QOL). Providing the exact meaning of the term "QOL" is rather difficult because an elaborate theoretical framework is (still) lacking. Therefore, there are nearly as many definitions of QOL as there are studies on this subject [19]. Research suggests that poor perceived present quality of life (QOL) may be the most significant factor in psychological illness and stress related disorders in refugee populations [20].

Health-related quality of life (HRQOL) is a multidimensional concept that focuses on the impact health status has on quality of life and represents the subjective evaluation of physical, mental, emotional, and social functioning [21]. Other studies tried to analyze the quality of life of the immigrants, often using other questionnaires as a tool to measure it [22]. As we know, in a lot of studies concerning medicinal interventions, QOL has become an important endpoint. There is a connection between health and quality of life, i.e., better health means a better quality of life. The current study reveals good HRQOL among Afghan refugees. To the best of our knowledge, this study is the very first to assess the HRQOL of Afghan refugees in Pakistan.

## Methodology

### Study setting and design

The study design was a population-based "cross-sectional study" using a quantitative approach. The research was being carried out in Quetta, Pakistan. Some Afghan refugees were living in random public places such as "Killi Kamalo, Zamindar Colony, and Bashir Chowk," called secondary research sites, but the majority of Afghan refugees were found in "Hazara Town, Jungle Bagh, Ghausabad, and Saranan Camp," called main research sites. As a reliable sample, the majority of the data was collected from the main research sites, and some was collected randomly from secondary research sites.

#### Study sampling and study duration

The duration of the study was from March 2017 to September 2017. A time-based study was being carried out, and a convenient sampling was done. The data collected was reliable because it was collected only from those who agreed to participate in the study as were willing to fill out the questionnaire. Those who did not agree to participate were excluded from the study.

#### Study tool and statistical analysis

The EQ5D-3L questionnaire along with demographic characteristics were used (S1 File). All the participants were asked to fill out the EQ5D-3L questionnaire. The EQ5D-3L (Urdu language) questionnaire was used. It was a self-administered questionnaire, but due to a lack of education, many of the samples were helped out to fill the questionnaire. All three versions are available in a wide range of languages and modes of administration [23]. The EQ-5D description system, which consists of five dimensions of health—mobility, self-care, common activities, pain/discomfort, and anxiety/depression—is also included. Each of these categories has three possible answers. Within a specific EQ5D dimension, the response tracks three severity levels (no problems/some or moderate problems/extreme problems).

*Scoring the EQ-5D-3 L VAS*. The EQ scale of the visual analog scale (VAS) measures subjects’ self-reported health on a linear visual analog (vas scale ranging from 0 to 100, with terminals captioned ’The best conditions you could envision’ and ’The very worst health you can think. precisely, on the EQ- visual analog scale (VAS), “100” represents the finest health level in addition to it the “0” represents the most severe health situation possible.

The version we used was EQ-5D-3L because it was available in translated Urdu. The collected data was analyzed and interpreted by using SPSS (Statistical Package for Social Sciences) 22.0 version software [24], the results obtained were shown in the form of frequency tables. The descriptive statistical analysis to expound participants’ demographic characteristics and the inferential statistics (Kruskal-Wallis and Mann-Whitney test, P < 0.05) were used to compare EQ-5D scale scores.

The ethical approval was obtained from the research committee of the department of pharmacy practice, faculty of pharmacy and health sciences, with reference number DPP/SS/83/17, University of Baluchistan, Quetta, Pakistan. According to the ethical guidelines, the study participants provided informed consent (S1 File). Additionally, we also obtained consent from the parents or guardians of the participants (only in the case of minors’ participants).

## Results

The study sample consisted of 729 participants who took part in the study. Participants’ ages ranged from 12 to 98 years. Here the Table 1 reflects the demographic information of the participants studied. Majority of the respondents were from 21 to 31 year of age and the dominated 480 (65.8%) participated respondent were male, 450 (61.7%) participants were married with 441 (60.5%) had urban residency in Afghanistan. The majority of participants (90.1%) (n = 657) did not reside in any nation other than Pakistan, with 234 (32.1%) of them having lived there for at least ten years. The majority of respondents (n = 222) were from Kandahar, and 95.5% of the 696 participants lived with their families. Almost 42% (n = 306) had no education, and 33.7% (n = 246) had a private job. 294 (40.3%) had no monthly income, and 79.4% (n = 579) preferred to be treated in a hospital or Basic health unit (BHU). Most of the respondent were not arrested for a crime. Mobility (n = 654), self-care (n = 666) (91.4%), usual work (n = 594) (81.5%), pain and discomfort (n = 519) (71.2%), anxiety and depression (n = 450) (61.7%) were all indicated as not being a problem by the majority of respondents by using self-reporting EQ-5D as shown in Table 2. A total of 24 health conditions as shown in Table 3, the majority of respondents (55.6%) have 1111 health states and no health issues. The EQ-5D Health Index/Score is significantly associated with age (p = 0.001), marital status (p = 0.001), locality in Afghanistan (p = 0.008), year living as refugees (p = 0.001), living as a refugee somewhere other than Pakistan (p = 0.004), place of residence in Afghanistan (p = 0.018), education (p = 0.033), occupation (p = 0.001), and being arrested for crime (p = 0.005) as shown in Table 4. In Table 5, age (p = 0.001), marital status (p = 0.001), education (p = 0.003), occupation (p = 0.001), and arrest for crime (p = 0.041) are significantly associated with VAS score.

**Table 1:** Demographic characteristics of study respondents (n  =  729).

Description	Frequency	Percentage
**Age (years)**		
12 to 21	222	30.5
22 to 31	246	33.7
32 to 41	114	15.6
42 to 51	51	7.0
52 to 61	51	7.0
62 and above	45	6.2
**Gender**		
Male	480	65.8
Female	249	34.2
**Marital Status**		
Unmarried	279	38.3
Married	450	61.7
**Locality In Afghanistan**		
Urban	441	60.5
Rural	288	39.5
**Year Living as Refugee in Pakistan**		
2 to 10	111	15.2
11 to 20	234	32.1
21 to 30	198	27.2
31 and above	186	25.5
**Live as Refugee other than Pakistan**		
Yes	72	9.9
No	657	90.1
**Place of residence in Afghanistan**		
Kabul	66	9.1
Jalalabad	57	7.8
Kandahar	222	10.5
Mazar Sharif	63	8.6
Herat	36	4.9
Sayyad/Sar-e-pol	54	7.4
Zabul	30	4.1
Baghlan	30	4.1
Helmand	21	2.9
Ghazni	27	3.7
Pashmul	30	4.1
Faryab	18	2.5
Kunduz	75	10.3
**Living Status**		
Alone	33	4.5
With Family	696	95.5
**Education**		
No Education	306	42
Religious	129	17.7
Primary	72	9.9
Middle	66	9.1
Matric	39	5.3
Inter	45	6.2
Graduation	60	8.2
Others	12	1.6
**Occupation**		
Unemployed	45	6.2
Private Job	246	33.7
Own Business	153	21
Student	87	11.9
Housewife	174	23.9
Others	24	3.3
**Monthly Income**		
No income	294	40.3
Less than 10,000	138	18.9
10,000 to 20,000	189	25.9
20,001 to 30,000	51	7.0
More than 30,000	57	7.8
**Preferred place of Treatment**		
Hospital/BHU	579	79.4
General practitioner	81	11.1
Traditional healer	69	9.5
**Arrested for crime**		
Yes	39	5.3
No	690	94.7

**Table 2:** Self-reported health status (EQ-5D).

EQ-5D Domain	Frequency	Percentage
**First Domain (Mobility)**		
No Problem in walking about	654	89.7
Some Problem in Walking about	75	10.3
Confined to bed	0	0
**Second Domain (Self-care)**		
No Problem in self-care	666	91.4
Some Problem in washing and dressing myself	57	7.8
Unable to wash and dress myself	6	8
**Third Domain (Usual Work)**		
No Problem in performing usual activities	594	81.5
Some Problems in performing usual activities	123	16.9
Unable to perform usual activities	12	1.6
**Forth Domain (Pain and Discomfort)**		
No pain and discomfort	519	71.2
Some pain and discomfort	195	26.7
Extreme pain and discomfort	15	2.1
**Fifth Domain (Anxiety and Depression)**		
Not anxious or depress	450	61.7
Moderately anxious or depress	267	36.6
Extremely anxious or depress	12	1.6

**Table 3:** Frequency of self-reported (EQ-5D) health states.

S No	EQ-5D states	Frequency	Percentage
1	11111	405	55.6
2	11112	90	12.3
3	11113	6	0.8
4	11121	12	1.6
5	11122	57	7.8
6	11132	3	0.4
7	11211	3	0.4
8	11221	6	0.8
9	11222	45	6.2
10	11311	3	0.4
11	11331	3	0.4
12	12111	3	0.4
13	12122	3	0.4
14	12211	3	0.4
15	12222	3	0.4
16	12231	3	0.4
17	13333	6	0.8
18	21112	3	0.4
19	21121	3	0.4
20	21122	9	1.2
21	21211	3	0.4
22	21221	3	0.4
23	21222	12	1.6
24	22222	42	5.8

**Table 4:** Different variables EQ-5D Score (0.85 ± 0.20).

Description	Frequency	Mean ± SD	P value
Age* (years)			
12 to 21	222	0.95 ± 0.10	**0.001**
22 to 31	246	0.86 ± 0.23	
32 to 41	114	0.90 ± 0.12	
42 to 51	51	0.77 ± 0.19	
52 to 61	51	0.16 ± 0.15	
62 and above	45	0.60 ± 0.29	
**Gender** **			
Male	480	0.86 ± 0.22	0.082
Female	249	0.84 ± 0.18	
**Marital Status** **			
Unmarried	279	0.89 ± 0.24	**0.001**
Married	450	0.83 ± 0.17	
**Locality in Afghanistan** **			
Urban	441	0.87 ± 0.20	**0.008**
Rural	288	0.82 ± 0.21	
**Year Living as Refugee** *			
2 to 10	111	0.93 ± 0.21	**0.001**
11 to 20	234	0.87 ± 0.17	
21 to 30	198	0.86 ± 0.17	
31 and above	186	0.77 ± 0.25	
**Live As Refugee other than Pakistan** **			
Yes	72	0.69 ± 0.34	**0.004**
No	657	0.87 ± 0.18	
**Place of residence in Afghanistan** *			
Kabul	66	0.72 ± 0.35	**0.018**
Jalalabad	57	0.91 ± 0.13	
Kandahar	222	0.89 ± 0.16	
Mazar Sharif	63	0.88 ± 0.15	
Herat	36	0.90 ± 0.12	
Sayyad/Sar-e-pol	54	0.92 ± 0.13	
Zabul	30	0.81 ± 0.29	
Baghlan	30	0.69 ± 0.17	
Helmand	21	0.84 ± 0.12	
Ghazni	27	0.78 ± .25	
Pashmul	30	0.94 ± .12	
Faryab	18	0.92 ± .11	
Kunduz	75	0.85 ± .26	
**Living Status** **			
Alone	33	0.68 ± 0.40	0.083
With Family	696	0.86 ± 0.19	
**Education** *			
No Education	306	0.84 ± 0.16	**0.033**
Religious	129	0.81 ± 0.20	
Primary	72	0.89 ± 0.14	
Middle	66	0.89 ± 0.28	
Matric	39	0.87 ± 0.17	
Inter	45	0.84 ± 0.26	
Graduation	60	0.96 ± 0.06	
Others	12	0.60 ± 0.60	
**Occupation** *			
Unemployed	45	0.79 ± 0.20	**0.001**
Private Job	246	0.89 ± 0.18	
Own Business	153	0.80 ± 0.26	
Student	87	0.97 ± 0.06	
Housewife	174	0.82 ± 0.16	
Others	24	0.66 ± 0.36	
**Monthly Income** *			
No income	294	0.84 ± 0.19	0.095
Less than 10,000	138	0.88 ± 0.29	
10,000 to 20,000	189	0.85 ± 0.19	
20,001 to 30,000	51	0.84 ± 0.15	
More than 30,000	57	0.88 ± 0.14	
**Preferred place of Treatment** *			
Hospital/BHU	579	0.86 ± 0.21	0.372
General practitioner	81	0.82 ± 0.21	
Traditional healer	69	0.84 ± 0.17	
**Arrested for crime** **			
Yes	39	0.62 ± 0.42	**0.005**
No	690	0.87 ± 0.18	

* Kruskal Wallis Test.

** Mann Whitney Test.

**Table 5:** VAS Score (78.60 ± 11.10) among variables.

Description	Frequency	Mean ± SD	P value
Age* (years)			
12 to 21	222	83.45 ± 7.62	**0.001**
22 to 31	246	80.00 + 10.83	
32 to 41	114	77.76 + 9.56	
42 to 51	51	72.65 + 16.01	
52 to 61	51	70.00 + 9.84	
62 and above	45	65.67 + 7.28	
**Gender** **			
Male	480	78.84 + 10.89	0.597
Female	249	78.13 + 11.54	
**Marital Status** **			
Unmarried	279	82.10 + 10.74	**0.001**
Married	450	76.43 + 10.79	
**Locality In Afghanistan** **			
Urban	441	79.73 + 10.73	0.057
Rural	288	76.88 + 11.47	
**Year Living as Refugee** *			
2 to 10	111	81.89 + 7.84	0.074
11 to 20	234	79.10 + 9.89	
21 to 30	198	78.86 + 11.56	
31 and above	186	75.73 + 13.08	
**Live As Refugee other than Pakistan** **			
Yes	72	80.63 + 11.45	0.320
No	657	78.38 + 11.06	
**Place of residence in Afghanistan** *			
Kabul	66	75.68 + 12.27	0.323
Jalalabad	57	81.32 + 10.78	
Kandahar	222	78.85 + 10.74	
Mazar Sharif	63	76.19 + 7.73	
Herat	36	78.75 + 8.01	
Sayyad/Sar-e-pol	54	78.89 + 8.14	
Zabul	30	80.00 + 12.69	
Baghlan	30	70.00 + 14.72	
Helmand	21	76.43 + 12.48	
Ghazni	27	85.00 + 8.66	
Pashmul	30	82.00 + 10.59	
Faryab	18	80.93 + 10.68	
Kunduz	75	79.40 + 14.16	
**Living Status** **			
Alone	33	79.55 + 13.68	0.569
With Family	696	78.56 + 10.98	
**Education** *			
No Education	306	76.52 + 10.11	**0.003**
Religious	129	78.72 + 10.86	
Primary	72	79.58 + 9.88	
Middle	66	82.73 + 10.88	
Matric	39	78.85 + 12.10	
Inter	45	77.33 + 13.47	
Graduation	60	86.00 + 11.53	
Others	12	68.75 + 1436	
**Occupation** *			
Unemployed	45	74.33 + 13.99	**0.001**
Private Job	246	79.02 + 9.07	
Own Business	153	78.73 + 12.48	
Student	87	86.03 + 8.27	
Housewife	174	76.03 + 10.95	
Others	24	73.13 + 11.10	
**Monthly Income** *			
No income	294	77.96 + 12.13	0.966
Less than 10,000	138	79.57 + 9.59	
10,000 to 20,000	189	78.81 + 10.65	
20,001 to 30,000	51	79.41 + 9.66	
More than 30,000	57	78.16 + 12.38	
**Preferred place of Treatment** *			
Hospital/BHU	579	79.30 + 10.41	0.089
General practitioner	81	77.41 + 14.36	
Traditional healer	69	74.13 + 11.74	
**Arrested for crime** **			
Yes	39	71.92 + 11.99	**0.041**
No	690	78.98 + 10.95	

* Kruskal Wallis Test

** Mann Whitney Test.

## Discussion

The current study reveals good HRQOL among Afghan refugees. Keeping in mind that the descriptive score was a little bit higher than the perceived health status, this indicates that the actual health condition is better than what was perceived by the participants. To the best of our knowledge, this study is the very first to access the HRQoL of Afghan refugees in Pakistan The HRQOL of the relatively young adult Palestinian refugee population was relatively good for physical health, psychological health, and social relations, but was poor for environment, as the study was conducted in Jordan about the health-related quality of life of Palestinian refugees [21].The finding were again supported by the study QOL among Syrian refugees in Kurdistan which falls largely within a range of QOL scores [20]. This is consistent with earlier studies of the general population [15], immigrants in Sweden [25], torture survivors among refugees in Denmark [26], and a recently released study among Syrian refugees [27]. In contrast the Northern Greece refugees showed poor HRQL [28].

The current study was done with the EQ-5D instrument, which has five dimensions to determine the health status of the participants, including mobility, self-care, usual activities, pain or discomfort, and anxiety or depression. Each of the five dimensions is divided into three levels of problem perception. The current study included a total of 24 health statuses, with the most dominant EQ-5D status having no problems in all five domains. The current study findings are also in line with what is reported in a study done in the Netherlands, which reported that Afghan refugees considered their physical and mental health to be good [2]. In comparison to the current study in the fifth domain, where the majority of respondents were not depressed, two different studies elsewhere in the world reported that distress symptoms occur at higher rates in the US and Afghans residing in Turkey are at high risk of psychopathology [29, 30]. There are many factors that influence the health of Afghan refugees. As we know, resettlement in a new culture may lead to many health problems. Health may be affected by starting your life in a country with different culture, religion, language, social structure, values, and environment, as well as by the behavior of the people where refugees are resettled. The areas of Pakistan where Afghan refugees are living have the same culture and religion. As we know, the majority of the Afghans living in Quetta are Pashtuns [17], and the residing people in Quetta are also of the same ethnicity having same values, environment, and social structure- even the dressing is same. Pashtunistan, the land of the Pashtuns or Pathans, lies on both sides of the Durand Line, the frontier between Pakistan and Afghanistan. Since 1947, Afghanistan has consistently repudiated the Durand Line and demanded the right of self-determination for the Pashtuns because she does not consider them a part of Pakistan [31], and the Pashtun areas of Balochistan, including Quetta, also believe in the ideology of Pashtunistan and behave like brothers with Afghan refugees.

HRQOL had a significant relationship with many of the demographic characteristics in our study like age, marital status, locality in Afghanistan, living as a refugee somewhere other than Pakistan, year of Living as a refugee, place of residence in Afghanistan, education, occupation and being arrested for crime. There are mixed results when our findings are compared with those of other studies of the same nature. A study conducted in Italy reported that age was influential variable on HRQOL among refugees and asylum seekers using the SF-36 tool [22]. A study done using WHOQOL-BREF reported that age is significantly associated with physical, social and environmental domains [32]. Gerritsen et al. found that age, marital status, education, and year as refugees in that country were all significant factors [2]. A study mentioned that immigration background, gender, and age are significantly associated with physical and mental health [33]. Demographic factors such as employment status and income were found to be significantly related to the mental health of Afghan refugees living in Istanbul [30]. Although the majority of the respondents were not arrested for any crime. Beside the crime, if someone has done something wrong, authorities should act against them, but targeting someone only because he is Afghan isn’t fair [34]. It can be a reason that some of the respondents had problems in the psychological domain and may be age -dependent. A greater HRQOL is achieved by taking into account factors like similarity in culture, religion, language, social structure, values, and surroundings, as well as how people treat refugees.

### Study strengths and weaknesses

The majority of the refugee camps in the Quetta region were covered by the survey, which was done in the Balochistan province’s capital city. which will assist the government and policymakers in improving the HRQOL of Afghan refugees living in these camps. However, because we employ convenience sampling, which may limit the study’s generalizability, we are unable to generalize these findings to all Afghan refugees in the country as a whole.

## Conclusion

The current study reveals better HRQOL among refugees in Quetta, Pakistan. Results may vary elsewhere in Baloch areas and Panjab, Pakistan, due to changes in culture, language, social structure, values, and environmental conditions. Our study concluded that demographic characteristics like gender, living status, monthly income, and preferred place of treatment are not associated with health-related quality of life among Afghan refugees; however, the effect varies with age, marital status, locality in Afghanistan, year living as refugees, living as a refugee somewhere other than Pakistan, place of residence in Afghanistan, education, occupation, and being arrested for crime, which were significantly associated with the EQ-5D Health Index/Score. This study provides a baseline assessment for the health status of Afghan refugees and the results could be applied in improving HRQOL. It can improve more by improving the educational status and environmental variable such as income, which can result in better HRQOL. Future research should attempt to address educational issues in relation to the quality of life of refugees, as the identification of educational issues may assist in the design of educational-specific interventions in self-sustenance and improving quality of life.

## Supporting information

S1 File (PDF)

## References

[1] Shacknove AE. Who is a Refugee? Ethics. 1985;95(2):274–84.

[2] Gerritsen AA, Bramsen I, Devillé W, Van Willigen LH, Hovens JE, Van Der Ploeg HM. Physical and mental health of Afghan, Iranian and Somali asylum seekers and refugees living in the Netherlands. Social psychiatry and psychiatric epidemiology. 2006;41(1):18–26. doi: 10.1007/s00127-005-0003-5 16341619

[3] Tinghög P, Malm A, Arwidson C, Sigvardsdotter E, Lundin A, Saboonchi F. Prevalence of mental ill health, traumas and postmigration stress among refugees from Syria resettled in Sweden after 2011: a population-based survey. BMJ open. 2017;7(12):e018899. doi: 10.1136/bmjopen-2017-018899 29289940 PMC5778338

[4] Sigvardsdotter E, Vaez M, Hedman A-M, Saboonchi F. Prevalence of torture and other war-related traumatic events in forced migrants: a systematic review. Journal on Rehabilitation of Torture Victims and Prevention of Torture. 2016;26(2):41–73. 27858780

[5] Bogic M, Njoku A, Priebe S. Long-term mental health of war-refugees: a systematic literature review. BMC international health and human rights. 2015;15(1):1–41. 26510473 10.1186/s12914-015-0064-9 PMC4624599

[6] Steel Z, Chey T, Silove D, Marnane C, Bryant RA, Van Ommeren M. Association of torture and other potentially traumatic events with mental health outcomes among populations exposed to mass conflict and displacement: a systematic review and meta-analysis. Jama. 2009;302(5):537–49. doi: 10.1001/jama.2009.1132 19654388

[7] Assembly UG. Universal declaration of human rights. UN General Assembly. 1948.

[8] Jastram K, Achiron M. Refugee protection: a guide to international refugee law. 2001.

[9] Zieck M. The legal status of Afghan refugees in Pakistan, a story of eight agreements and two suppressed premises. International Journal of Refugee Law. 2008;20(2):253–72.

[10] Jacobsen K. Factors influencing the policy responses of host governments to mass refugee influxes. International Migration Review. 1996:655–78. 12292015

[11] Ahmed A. US provides $60m in 2022 for Afghan refugees in Pakistan. DAWN. 2023 6 jan 2023.

[12] Kronenfeld DA. Afghan refugees in Pakistan: not all refugees, not always in Pakistan, not necessarily Afghan? Journal of Refugee Studies. 2008;21(1):43–63.

[13] Lipson JG, Omidian PA. Health issues of Afghan refugees in California. Western Journal of Medicine. 1992;157(3):271. 1413768 PMC1011275

[14] Lipson JG. Afghan refugees in California: Mental health issues. Issues in mental health nursing. 1993;14(4):411–23. doi: 10.3109/01612849309006903 8244691

[15] Gariepy G, Honkaniemi H, Quesnel-Vallee A. Social support and protection from depression: systematic review of current findings in Western countries. The British Journal of Psychiatry. 2016;209(4):284–93. doi: 10.1192/bjp.bp.115.169094 27445355

[16] Quddus A, Luby SP, Jamal Z, Jafar T. Prevalence of hepatitis B among Afghan refugees living in Balochistan, Pakistan. International Journal of Infectious Diseases. 2006;10(3):242–7. 16448838 10.1016/j.ijid.2005.04.007

[17] Linkages C-B. AFGHANS IN QUETTA.

[18] Naeem F, Mufti KA, Ayub M, Haroon A, Saifi F, Qureshi SM, et al. Psychiatric morbidity among Afghan refugees in Peshawar, Pakistan. Children. 2005;429(29.9):14.8. 16092644

[19] De Vries J, Van Heck G. The assessment of quality of life in refugees. Quality of life assessment: International perspectives: Springer; 1994. p. 161–75.

[20] Aziz IA, Hutchinson CV, Maltby J. Quality of life of Syrian refugees living in camps in the Kurdistan Region of Iraq. PeerJ. 2014;2:e670. doi: 10.7717/peerj.670 25401057 PMC4230548

[21] Alduraidi H, Waters CM. Health-related Quality of Life of Palestinian Refugees Inside and Outside Camps in Jordan. Nursing Outlook. 2017. doi: 10.1016/j.outlook.2017.05.007 28622883

[22] Nante N, Gialluca L, De Corso M, Troiano G, Verzuri A, Messina G. Quality of life in refugees and asylum seekers in Italy: a pilot study. Annali dell’Istituto superiore di sanita. 2016;52(3):424–7. doi: 10.4415/ANN_16_03_14 27698301

[23] EQ-5D. EQ-5D Instruments: EQ-5D; 2017. Available from: https://euroqol.org/eq-5d-instruments/.

[24] Corp I. IBM SPSS statistics for windows, version 22.0. Armonk, NY: IBM Corp. 2013.

[25] Ranjbar V, Fornazar R, Ascher H, Ekberg‐Jansson A, Hensing G. Physical and mental health inequalities between native and immigrant Swedes. International Migration. 2017;55(2):80–96.

[26] Carlsson JM, Olsen DR, Mortensen EL, Kastrup M. Mental health and health-related quality of life: a 10-year follow-up of tortured refugees. The Journal of nervous and mental disease. 2006;194(10):725–31. doi: 10.1097/01.nmd.0000243079.52138.b7 17041283

[27] Gottvall M, Sjölund S, Arwidson C, Saboonchi F. Health-related quality of life among Syrian refugees resettled in Sweden. Quality of Life Research. 2020;29:505–14. doi: 10.1007/s11136-019-02323-5 31617059 PMC6994443

[28] Beza S, Mavrodi AG, Kyratso G, Aletras VH. Health-Related Quality of Life Among Refugees and Asylum Seekers in Northern Greece. Journal of Immigrant and Minority Health. 2021:1–8. doi: 10.1007/s10903-021-01199-3 33830398

[29] Alemi Q, James S, Siddiq H, Montgomery S. Correlates and predictors of psychological distress among Afghan refugees in San Diego County. International journal of culture and mental health. 2015;8(3):274–88. doi: 10.1080/17542863.2015.1006647 26543500 PMC4629859

[30] Alemi Q, Stempel C, Baek K, Lares L, Villa P, Danis D, et al. Impact of Postmigration Living Difficulties on the Mental Health of Afghan Migrants Residing in Istanbul. International Journal of Population Research. 2016;2016.

[31] Qureshi SM. Pakhtunistan: The Frontier Dispute Between Afghanistan and Pakistan. Pacific Affairs. 1966;39(1/2):99–114.

[32] Abuawad MS. Assessing quality of life of Palestinian diabetic patients; refugees and non-refugees.

[33] Nesterko Y, Braehler E, Grande G, Glaesmer H. Life satisfaction and health-related quality of life in immigrants and native-born Germans: the role of immigration-related factors. Quality of Life Research. 2013;22(5):1005–13. doi: 10.1007/s11136-012-0239-y 22843126

[34] Sadaqat Jan PA. Pashtuns Claim Ethnic Profiling During Pakistan Extremism Crackdown. VOA. March 2017.

