# Supplementary material for: Assessment of health-related quality of life among Afghan refugees in Quetta, Pakistan
Source: PLoS One. 2024 Feb 1;19(2):e0288834. doi: 10.1371/journal.pone.0288834 (PMC10833568; doi:10.1371/journal.pone.0288834)
Supplement: S1 File — (PDF) [file pone.0288834.s001.pdf]

Annex 1: Study Questionnaire

"Assessment of health related quality of life among afghan refugees in Quetta Pakistan"

"رضامندی نامہ"

• یہ مطالعہ شعبہ فارمیسی فائنل ائر کے طالب علم شعیب کلیم کی جانب سے منعقد کی جارہی ہے جس سے افغان پناہ گزینوں کے صحت کے معیار کا اندازہ لگے گا۔

آپ کو اس میں شرکت کرنے کی درخواست کی جاتی ہے، آپ کی شرکت رضا کارانہ ہے آپ کسی بھی وقت مطالعہ چھوڑ سکتے ہیں آپ کے تمام معلومات کو صیغہ راز میں رکھا جائیگا۔

دستخط:۔ ____________

تاریخ: ____________

"ذاتی معلومات"

عمر ____________ جنس: ☐ مرد ☐ عورت

ازدواجی حیثیت: ☐ غیر شادی شدہ ☐ شادی شدہ

افغانستان میں سکونت: ☐ شہری ☐ دیہی پناہ گزین کی حیثیت سے گزارے گئے سال ______

پاکستان کے علاوہ دوسرے کسی دوسرے ملک میں پناہ گزین کی حیثیت سے: ☐ ہاں ☐ نہیں

افغانستان میں رہنے کی جگہ ____________

رہائش: ☐ اکیلے ☐ خاندان کے ساتھ

تعلیمی معیار: ☐ غیر تعلیم یافتہ ☐ مذہبی تعلیم ☐ پرائمری ☐ مڈل

☐ میٹرک ☐ انٹرمیڈیٹ ☐ گریجویٹ ☐ دیگر تعلیم

پیشہ: ☐ بے روزگار ☐ پرائیوٹ ملازم ☐ ذاتی کاروبار

☐ طالب علم ☐ خاتون خانہ ☐ دیگر

ماہانہ آمدنی: ☐ کوئی آمدنی نہیں ☐ 10000 سے کم ☐ 10000 سے 20000

☐ 20000 سے 30000 ☐ 30000 سے زیادہ

علاج کی منتخبہ جگہ: ☐ ہسپتال / بنیادی مرکز صحت ☐ عمومی معالج ☐ روایتی علاج

کیا آپ کو کبھی کسی جرم گرفتار کیا گیا ہے؟ ☐ ہاں ☐ نہیں

مندرجہ ذیل ہر گروپ کے کسی ایک خانے پر نشان ☑ لگائیے۔ برائے مہربانی ان جملوں کی نشاندہی کیجئے جو آپ کی آج کل کی صحت کی بہترین عکاسی کرتے ہیں۔

چلنا پھرنا

- ☐ مجھے چلنے پھرنے میں کوئی مشکل نہیں ہے
- ☐ مجھے چلنے پھرنے میں کچھ مشکل ہوتی ہے
- ☐ میں بالکل چل پھر نہیں سکتا/سکتی ہوں

اپنی دیکھ بھال کرنا

- ☐ مجھے اپنی دیکھ بھال کرنے میں کوئی مشکل نہیں ہے
- ☐ مجھے نہانے اور کپڑے پہننے میں کچھ مشکل ہوتی ہے
- ☐ میں خود نہا یا کپڑے نہیں پہن سکتا/سکتی ہوں

روزمرہ کے کام کاج *(مثلاً کام، پڑھائی، گھریلو کام کاج، خاندانی اور تفریحی مصروفیات)*

- ☐ مجھے اپنے روزمرہ کے کام کاج میں کوئی مشکل نہیں ہوتی ہے
- ☐ مجھے اپنے روزمرہ کے کام کاج میں کچھ مشکل ہوتی ہے
- ☐ میں اپنے روزمرہ کے کام کاج نہیں کر سکتا /سکتی ہوں

درد / بے آرامی

- ☐ مجھے کوئی درد یا بے آرامی نہیں ہے
- ☐ مجھے کچھ درد یا بے آرامی ہے
- ☐ مجھے شدید درد یا بے آرامی ہے

بے چینی/ ذہنی پریشانی (ٹینشن)

- ☐ مجھے کوئی بے چینی یا ذہنی پریشانی نہیں ہے
- ☐ مجھے کچھ بے چینی یا ذہنی پریشانی ہے
- ☐ مجھے شدید بے چینی یا ذہنی پریشانی ہے

بہترین

صحت

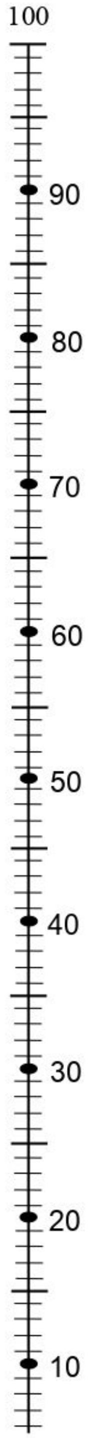

بدترین

صحت

اپنی آج کل کی صحت کی صحیح طور پر نشاندھی کرنے کے لیے ہم نے آپ کی آسانی کے لیے ایک (تھرماميٹر کی طرح) پیمانہ بنایا ہے۔ اس پیمانے پر سو (100) آپ کی بہترین اور صفر (0) آپ کی بدترین صحت کی نشاندھی کرتا ہے۔

ہم چاہیں گے کہ آپ اس پیمانے پر نشاندھی کریں کہ آج کل آپ کے خیال میں آپ کی صحت کیسی ہے (بہترین یا بدترین) نیچے دئے گئے (آپ کی آج کل کی صحت) خانے سے ایک لائن لگائیں جو پیمانے پر موجوداس نشان تک ہو جو آپ کی آج کل کی صحت کی صحیح نشاندھی کرتی ہو۔

آپ کی آج کل کی صحت
