# Supplementary material for: Assessment of health-related quality of life among Afghan refugees in Quetta, Pakistan
Source: PLoS One. 2024 Feb 1;19(2):e0288834. doi: 10.1371/journal.pone.0288834 (PMC10833568; doi:10.1371/journal.pone.0288834)
Supplement: Attachment — Submitted filename: Reviewer 02 Comments.docx [file pone.0288834.s002.docx]

**Reviewer 02 Comments**

To be accepted for publication the written English must be improved within the methods, results and discussion sections. The language is currently not at a high enough standard for publication. E.g. there are capital letter appearing in the middle of sentences when they are not warranted.

The formatting of the tables is a problem (rows don't align) and need to be re-formatted to make it easier to interpret. The tables are not well described in the titles, which seem to have random numbers inserted within the titles without any description.

**Response**

The manuscript has been checked thoroughly, and capital letters have been removed. The format of the tables changed as per suggestions.
