# Supplementary material for: Assessment of health-related quality of life among Afghan refugees in Quetta, Pakistan
Source: PLoS One. 2024 Feb 1;19(2):e0288834. doi: 10.1371/journal.pone.0288834 (PMC10833568; doi:10.1371/journal.pone.0288834)
Supplement: Attachment — Submitted filename: Reviewer 3 comments and response from authors.docx [file pone.0288834.s003.docx]

**Reviewer #3 comments and response from authors**

**Comment**

It is recommended to perform multivariate analysis (regression).

**Response:**

Thanks for your recommendation, we performed the multivariate regression analysis, but the results were not so satisfied to include in the manuscript and the reasons are:

1. We employed non-parametric test for our analysis, because of non-linear relationship and Regression analysis assumes a linear relationship between variables.
2. For certain research objectives, such as exploratory or descriptive studies, regression analysis may not be applicable. Regression might not add much value to the study in certain circumstances; alternative methods of analysis may be more suited as we did in our manuscript to report the solid results.

**Comment**
• Is the Urdu version of the EQ5D-3Lquestionnaire valid and reliable? Please provide a reference. And do all Afghan refugees in Pakistan have the ability to understand Urdu? Did you get the data through interviews?

**Response:**

Yes, it is valid and reliable and we also mentioned the reference in methodology as reference number 22. By clicking that reference 22, everyone can easily find the EQ-5D-3L questionnaires in many different languages including Urdu. Yes, majority can speak and understand Urdu language. We did the data collection by asking them question only if they were having any problem in understating the EQ5D questionnaire.

**Comment**
• In the method field, write the name of the analysis used.

**Response:**

Thanks, it has been modified and mentioned the analysis used in the study.

**Comment**
• In the discussion section, write the strengths and weaknesses of the study.
• And keep in mind that your study does not have external validity because sampling was available and cannot be generalized to the Afghan refugee community in Pakistan.

**Response:**

Thanks! We added the strengths and weaknesses section to the discussion part of the manuscript. We also mentioned that the results can’t be generalized to all Afghan refugees.
